# Micronutrients and socio-demographic factors were major predictors of anaemia among the Ethiopian population

**DOI:** 10.1017/S0007114523001472

**Published:** 2023-12-28

**Authors:** Adamu Belay, Edward J. M. Joy, R. Murray Lark, E. Louise Ander, Scott D. Young, Elizabeth H. Bailey, Martin R. Broadley, Dawd Gashu

**Affiliations:** 1 Center for Food Science and Nutrition, Addis Ababa University, Addis Ababa, Ethiopia; 2 Food Science and Nutrition Research Directorate, Ethiopian Public Health Institute, Gulele Sub City, Addis Ababa, Ethiopia; 3 Faculty of Epidemiology and Population Health, London School of Hygiene and Tropical Medicine, London, UK; 4 School of Biosciences, University of Nottingham, Sutton Bonington Campus, Loughborough, Leicestershire, UK; 5 Rothamsted Research, West Common, Harpenden, Hertfordshire, UK

**Keywords:** Anaemia, Demographic groups, Hemoglobin, Risk factors, Selenium, Zinc

## Abstract

Anaemia is characterised by low hemoglobin (Hb) concentration. Despite being a public health concern in Ethiopia, the role of micronutrients and non-nutritional factors as a determinant of Hb concentrations has been inadequately explored. This study focused on the assessment of serum micronutrient and Hb concentrations and a range of non-nutritional factors, to evaluate their associations with the risk of anaemia among the Ethiopian population (*n* 2046). It also explored the mediation effect of Zn on the relation between se and Hb. Bivariate and multivariate regression analyses were performed to identify the relationship between serum micronutrients concentration, inflammation biomarkers, nutritional status, presence of parasitic infection and socio-demographic factors with Hb concentration (*n* 2046). Sobel–Goodman test was applied to investigate the mediation of Zn on relations between serum se and Hb. In total, 18·6 % of participants were anaemic, 5·8 % had iron deficiency (ID), 2·6 % had ID anaemia and 0·6 % had tissue ID. Younger age, household head illiteracy and low serum concentrations of ferritin, Co, Cu and folate were associated with anaemia. Serum se had an indirect effect that was mediated by Zn, with a significant effect of se on Zn (*P* < 0·001) and Zn on Hb (*P* < 0·001). The findings of this study suggest the need for designing a multi-sectorial intervention to address anaemia based on demographic group.

Low Hb concentrations in human are an indicator of anaemia, which is often linked with iron deficiency (ID)^(1)^. ID may be the most widely spread micronutrient deficiency, globally^(2)^. Previous estimates of anaemia in various age/sex categories by country between 1960 and 1983^(3)^ have suggested that ID contributes half of anaemia cause worldwide^(4)^, although more recent estimates (between 1995 and 2011) indicated a lower proportion, particularly in contexts with high prevalence of anaemia and infections^(4)^. Anaemia can affect children’s physical and mental development and can lead to greater infection risk and a decrease in work capacity due to fatigue in adults. The cause of low Hb is multi-factorial, with nutritional (vitamins and minerals) and non-nutritional risk factors (infection, blood loss, pregnancy) being the most recognised^(1,5)^. In sub-Saharan Africa investigating the potential role of these risk factors when interpreting Hb concentration in large-scale surveys is warranted.

The Ethiopian National Micronutrient Survey (ENMS) was conducted in 2015 across several demographic groups. Micronutrient biomarkers, socio-demography, presence of parasitic infection, anthropometry and dietary measurements were included during the survey^(6)^. Concentrations of Zn, se, folate and other mineral micronutrients were further analysed in archived serum from ENMS. In addition, Fe status of the studied population was determined.

Among several mineral micronutrients, Ca, Co, Cu, Mg, Mo, se and Zn are evidenced to be linked with haematological parameters^(7)^. Most of the mineral micronutrients are involved during haematopoiesis via the metabolically important enzymatic pathways^(7,8)^. Several studies have shown that serum Zn concentration may independently affect Hb, regardless of Fe status^(8,9)^. Zn may affect Hb via several Zn-dependent enzymes involved in Hb synthesis^(10)^ and erythropoiesis stimulation^(11)^.

Serum se has also been found to positively associated with Hb in different demographic groups in the UK^(12)^, USA^(13)^, Vietnam^(14–16)^, northeast Brazil^(17)^ and New Zealand^(8)^. Possible mechanisms whereby low se status could potentially contribute to low Hb concentrations include the role of se as antioxidant in erythrocytes^(18)^ and in the protection of erythrocytes from inﬂammation^(19)^. On the relation between serum se and Zn, studies revealed that the low serum se concentrations might have the potential to compromise Zn status^(20,21)^ and as a consequence may have an indirect negative impact on Hb concentration. The mediation effect of Zn on the relation between serum se and Hb uses to understand the mechanisms by which how Zn status is affected^(21)^ and provide a new insight for the scientific community to consider the relationship among these important micronutrients and Hb while considering interventions.

Association of Mg with Hb is illustrated in other studies in which Mg deficiency in both animals and humans can result in inflammation and this in turn increases haemolysis^(22–25)^. Also, Ca may be associated with Hb: experimental studies showed that supplemental Ca does not interfere with the absorption of Fe if the dose of Ca does not exceeded 800 mg^(26–28)^, but if the Ca dose exceeds 1000 mg, it interferes with Fe absorption and may lead to anaemia^(29)^. Studies have shown that high blood Cu level is associated with anaemia^(30,31)^. Co is involved in cobalamin (vitamin B_12_) synthesis, which is a crucial element of haematopoiesis and low blood Co concentration can lead to anaemia^(32)^. Other studies have shown an inverse relationship between Co and Hb^(33,34)^. A few studies have reported that an excess intake of Mo caused anaemia^(35,36)^.

In Ethiopia, even though anaemia is highly prevalent and its incidence is multifactorial, there are no studies at national level investigating the relationships between serum micronutrients, nutritional status, parasitic infection and low Hb concentrations or anaemia in different demographic groups, warranting further exploration to evaluate the role of micronutrients and other factors on anaemia incidence. In this study, we extend the research emerging from the 2015 ENMS to investigate the associations of Ca, Co, Cu, Mg, Mo, se and Zn, in addition to inﬂammation markers, nutritional status and non-nutritional factors (sex, age, household head literacy, residence, stool parasite and health status), folate with Hb and the risk of anaemia. We hypothesise that low concentrations of micronutrients in blood and non-nutritional factors are associated with low Hb concentrations and risk for anaemia among different demographic groups of Ethiopian population. We further explored whether serum Zn concentration mediates the association between se and Hb concentration.

## Material and methods

### Study design and population

In the ENMS, several demographic groups were sampled from nine regions and two administrative cities. The study population participating in the 2015 survey consisted of men aged 15–54 years, non-pregnant women of reproductive age (WRA) aged 15–49 years, school-age children (SAC) aged 6–14 years and young children (YC) aged 6–59 months. Details of the sampling strategy have been reported elsewhere^(6)^. The subjects were drawn from a random selection of 360 enumeration areas based on the 2007 Ethiopian population and housing census. Each enumeration area had on average 181 households (150–200)^(37)^. From each enumeration area, eleven households were selected randomly. The response rate was 94·5 %.

The present study used socio-demographic data, anthropometry, Hb concentration, parasitic infection, health status and inflammation biomarkers from the ENMS, and serum mineral micronutrient concentration in archived samples reported in subsequent studies^(6,38)^. Data were integrated using STATA software with a total of 2046 complete records considered for this study.

### Data collection and analysis

#### Socio-demography

Socio-demographic information was collected in the ENMS through a structured questionnaire. In the ENMS, data collectors and supervisors were trained on data collection and quality of data. There were forty-one data collectors trained in nutrition and public health, twenty-three supervisors with data collection experience, forty-six phlebotomists and twenty-three laboratory technicians who graduated in clinical laboratory, nursing and laboratory technology. The questionnaire was pilot tested^(6)^.

#### Collection, processing and analysis of serum mineral micronutrients

Blood collection and processing methods are described elsewhere^(6,38)^. Briefly, venous blood samples were taken from participants following the WHO blood collection guidelines^(39)^. Serum Zn and ferritin were adjusted using the Biomarkers Reflecting Inflammation and Nutritional Determinants of Anemia method, in cases of inflammation^(40)^. The time of last meal and the time of blood draw were recorded for all study subjects during blood collection.

The analysis of mineral micronutrients in serum samples is described in detail elsewhere^(41–43)^. Briefly, elemental concentrations in serum samples were determined using inductively coupled plasma-MS (Thermo Fisher Scientifc iCAPQ, Thermo Fisher Scientific) at University of Nottingham, UK, where the laboratory was accredited in all parameters except Mo. The limit of detection for all elements was measured as 3 × sd of 10 operational blanks; the limit of quantification was calculated as 10 × sd. Accuracy was verified with two Seronorm™ reference materials: L-1 (Lot 1801802) and L-2 (Lot 1801803) (Nycomed Pharma AS). These were prepared in an identical way to samples and calibration standards and typically run at the beginning and at the end of every analytical run. Average elemental recovery (%; *n* 24) was determined across ten analytical batches of blood serum, and the reference ranges are reported elsewhere^(41–43)^. Quality control samples for Fe and inflammatory markers (soluble transferrin receptor (sTfR), ferritin, *α*-1 acid glycoprotein (AGP), C-reactive protein (CRP)) were also analysed for every batch of samples and the results fell within the acceptable ranges. Serum CRP > 5 mg/l and AGP > 1 g/l were used to indicate the presence of acute infection and adjust ferritin values^(40,44)^. During the analysis, the inter- and intra-assay CV, respectively, for the serum samples (*n* 20) was 1·6 and 0·9 % for sTfR, 2·1 and 1·5 % for ferritin, 2·6 and 1·0 % for CRP, 1·7 and 1·5 % for AGP, 8 % and 4·4 % for Mg, 9 and 0·7 % for Ca 10 and 6·5 % for Co, 9 and 7·9 % for Cu, 7 and 2·3 % for Zn, 6 % and 0·9 % for se and 18 and 11·9 % for Mo.

#### Hb, Ferritin, sTfR, C-reactive protein, *α*-1 acid glycoprotein, stool and folate analysis

Hb concentration was measured in venous blood samples using a Hemocue® photometer in the field (Hb 201, Hemocue AB). The Hemocue HB 201+ analyser has an internal quality control (high, medium and low concentration). The CV of low (8 µg/dl), medium (11·8 µg/dl) and high (15·9 µg/dl) Hb concentration was 1·8, 1 and 0·6 %, respectively. The WHO recommendation cut-off values were used to define low Hb concentration (WHO 2001)^(45)^ on the basis of age and sex. The participant’s Hb concentration was adjusted for altitude following the method by Sullivan *et al*.^(46)^.

Ferritin, sTfR, CRP and AGP in serum samples were analysed using immunoturbidimetric method using Cobas 6000 (Roche kits Germen) instrument at an accredited Clinical Chemistry Laboratory at the Ethiopian Public Health Institute (EPHI). Stool samples were collected from participants. The stool collection kit containing 10 % formalin with integrated spoons and disposable gloves was given to the subjects followed by brief instruction on collection and handling of stool samples. Two bean-sized stool samples were placed into the stool cup. The stool containing cups were picked up the same or following day and given to trained laboratory technicians for analysis^(47)^. Just after collection, they were examined by clinical laboratory technicians in the field for presence of intestinal parasites using Lugol’s iodine to observe cysts of the intestinal protozoan parasites. Positive samples for the presence of intestinal parasites were kept in plastic tube containing 10 % formalin and transported to EPHI. The preserved stool samples were analysed with an ether sedimentation technique as described by Ritchie^(48)^ at parasitological laboratory, EPHI to identify the parasitic organisms.

Serum folate was assessed using a microbiologic assay. The methodology for preparation and analysis of serum samples for folate is described in detail elsewhere (Sisay *et al*. ^(49)^).

The largest demographic group was WRA, and serum folate concentration was determined for this group only (online Supplementary Table 1). The analytical assay inter- and intra-assay CV for serum folate was 8·9 and 4·1 %, respectively.

#### Nutritional assessment

Children ≤ 2 years and not able to stand by their own have been weighed in the arms of their mother or guardians using the mother–child tare function on the scale. Children > 2 years old and can stand stood on the centre of the weighing scale with the body weight evenly distributed between both feet and their weight was measured and recorded. Both young and older children had minimal clothing but without shoe during weight measurement. Weight was measured to the precision of 0·1 kg. Electronic digital scale (UNICEF SECA 874 U, UNICEF Supply Division) was used to measure the weight of participants. The same weighing scale was used to measure the weight of adult participants.

Recumbent length for ≤ 2 years old children and standing height for > 2 years participants was measured to the nearest 0·1 cm using a wooden all age convertible stadiometer (ShorrBoard ICA measuring board) with an upright wooden base and a movable headpiece. BMI (weight/height^2^ (kg/m^2^)) was calculated, and the threshold of 18·5 kg/m^2^ was used to identify wasting/thinness for women and men whose age is greater than 19 years, BMI 18·5 kg/m^2^ to 2 5 kg/m^2^ was considered normal while BMI greater than 25 kg/m^2^ was considered as overweight. Overweight (> 1 s d of BMI-for-age z-score), thinness/wasting (< –2 sd of BMI-for-age z-score) and normal (> –2 sd but ≤ 1 sd of BMI-for-age z-score) were defined for school children and adolescents using WHO Anthroplus Software Version 1.0.4 according to the WHO references^(50)^. For under-five children, anthropometric indices were calculated using Emergency Nutrition Assessment SMART 2011 software. Wasting and stunting were defined as < –2 sd of WAZ and < –2 sd of HAZ, respectively.

#### Biomarker cut-off

Prevalence of Zn deficiency among all demographic groups was determined following the recommendation by King *et al*.^(51)^. Serum Zn concentration < 65 µg/dl for morning, non-fasting samples for YC and SAC and also serum Zn < 57 µg/dl for afternoon, non-fasting samples were considered deficient. For children ≥ 10 years old, serum Zn concentrations < 70 µg/dl for males and < 66 µg/dl for females for morning, non-fasting samples were considered deficient. Furthermore, serum Zn concentrations < 61 µg/dl for male and < 59 µg/dl for females for afternoon, non-fasting samples were considered deficient. Cut-off of < 74 µg/dl and < 70 µg/dl serum Zn was used for men and < 70 µg/dl was used for non-pregnant WRA for morning, fasting samples. Furthermore, cut-off values of serum concentrations to define deficiency were 8·4 mg/dl for Ca, 0·022 μg/dl for Co, 75 μg/dl for Cu, 1·8 mg/dl for Mg, 0·02 μg/dl for Mo and 7 µg/dl for se^(52–54)^.

Hb concentration cut-offs to define anaemia were < 11·0 g/dl for YC < 5 years of age; < 11·5 g/dl for SAC 5–11 years; < 12·0 g/dl for SAC aged 12–14 years; < 12·0 g/dl for non-pregnant WRA and < 13·0 g/dl for men.

ID anaemia was defined as ID with low Hb concentration. ID is defined as serum ferritin (inflammation adjusted) < 12 µg/l for YC and < 15 µg/l for SAC, men and WRA^(45)^ or serum sTfR concentration > 4·4 mg/l for YC, SAC and WRA and > 5 mg/l for men. In addition, WRA with serum folate concentration < 6·8 nmol/l were considered as folate deficient^(55)^.

#### Statistical analyses

Descriptive statistical and multivariate regression analyses were computed using STATA (Version 14.0, StataCorp LLP). Survey weights were applied for descriptive statistics. Multivariate regression analyses were used to explore the association between nutritional (mineral micronutrients and serum folate, Fe biomarkers, anthropometric characteristics) and non-nutritional factors (socio-demographic factors, infection, intestinal parasites, child illness) with Hb concentration and anaemia occurrence. The independent variables used in the linear and logistic regression models include age, demographic status, BMI, stool parasite, educational status, WAZ, ferritin, serum Ca, Co, Cu, Mg, Mo, se and Zn concentration, and inﬂammation markers. Following multiple regressions, normal distribution for residuals was checked. To generate a valid estimation of exposure effects, only variables with *P*-value < 0·20 in the bivariate regression analysis were kept for multivariate regression analysis. Multicollinearity was computed and the variables with variance inflation factors < 2 were included and a multilevel binary logistic regression model was applied to identify the predictor of anaemia^(56)^. A mediation analysis was performed to examine direct and indirect relations (through Zn) between serum se and Hb concentrations, and their effects were estimated with the use of the Sobel–Goodman test^(57,58)^. For all statistical analyses, differences with *P* < 0·05 were considered significant.

#### Ethics

This study was conducted according to the guidelines laid down in the Helsinki Declaration, and all procedures involving human subjects were approved by the National Research Ethics Review Committee at the Ministry of Science and Technology, Ethiopia (Reference 3·10/433/06). Written informed consent and assent were obtained from all adult and child participants, respectively. This study was also approved by the Research Ethical Review Committee at the EPHI (Protocol EPHI-IRB-140–2018). Archived serum samples were transferred from storage at EPHI to the University of Nottingham, UK for analysis under a Material Transfer Agreement.

## Results

### Anaemia prevalence by demographic groups and regions

Complete data were available for 2046 subjects. Demographic characteristics of the ENMS participants are reported in previous papers^(39,41)^. High proportion of anaemia prevalence was observed in wasting subjects (23·8 %) compared with normal and overweight subjects (online Supplementary Table 2).

Among the regions, Addis Ababa residents had the largest median Hb concentration (13·8 g/dl), but lowest Hb concentration was observed among residents in Afar (12·7 g/dl) followed by Somali (12·8 g/dl) region (*P* < 0·001). Among the demographic groups, the lowest and highest median Hb concentrations were observed among YC (12 g/dl) and men (14·5 g/dl), respectively. Median Hb concentration was greater among urban residents than rural residents (13·6 g/dl *v*. 12·9 g/dl; *P* < 0·001) (Table 1).

**Table 1.** Median Hb concentration**s** and anaemia prevalence (%) among the Ethiopian population, 2015 (Median values)

Characteristics	*n*	Hb concentration (g/dl)		Anaemia prevalence
		Median	Q1, Q3	
Regions				
Addis Ababa	111	13·8	12·9, 14·5	5·7
Afar	160	12·7	12·1, 13·9	20·5
Amhara	343	13·0	12·1, 14·1	19·1
Benishangul-Gumuz	142	13·3	12·3, 14·0	17·6
Dire Dawa	111	13·1	12·2, 14·3	13·7
Gambela	115	12·9	12·2, 14·0	15·1
Harari	166	12·9	12·6, 14·1	15·2
Oromia	315	13·0	12·1, 14·1	21·1
SNNPR	208	13·4	12·3, 14·3	16·7
Somali	122	12·8	11·8, 13·8	26·0
Tigray	253	12·9	12·2, 13·9	13·2
National	2046	13·1	12·2, 13·9	18·6
Demographic group				
YC	338	12·0	11·1, 12·8	22·9
SAC	620	12·8	11·9, 13·6	25·1
Men	236	14·5	13·6, 15·3	18·4
WRA	852	13·4	12·7, 14·2	11·3
Residence				
Urban	555	13·6	12·7, 14·5	11·6
Rural	1491	12·9	12·0, 13·9	19·9
Education status				
Household head is literate	1042	13·3	12·5, 14·4	13·6
Household head is illiterate	1004	12·8	11·9, 13·8	23·2

Q1, 25th percentile; Q3, 75th percentile; YC, young children; SAC, school-age children; WRA, women of reproductive age.

The cut-offs used to define anaemia were Hb < 11·0 g/dl for young children; < 11·5 g/dl for children 5–11 years; < 12·0 g/dl for children 12–14 years; < 12·0 g/dl for WRA and < 13·0 g/dl for men; household heads attended elementary school or above were considered literate.

About 19 % of the population were anaemic. Among the regions, the highest anaemia prevalence was observed in Somali (26 %) followed by Oromia (21 %) and Afar (20·5 %). The lowest prevalence of anaemia was observed in Addis Ababa (5·7 %). SAC had highest prevalence of anaemia (25·1 %) followed by YC (22·9 %). Urban residents had lower anaemia prevalence than rural residents (11·6 % *v*. 19·9 %) (Table 1). As per the WHO classifications, anaemia was a moderate public health problem in YC and SAC, and a mild problem in WRA and men.

### Iron deficiency and iron deficiency anaemia prevalence by demographic group

Only 5·8 % of the participants had ID and 2·6 % had iron deficiency anaemia (IDA) (Table 2). Prevalence of ID ranged from 0 in the Amhara region to 14·6 % in Somali region. The prevalence of ID among YC and SAC was 13·8 and 3·3 %, respectively (Table 2). IDA was greater among YC than other demographic groups. Higher prevalence of ID and IDA was found among rural compared with urban residents. Greater ID was observed among participants from illiterate than literate household heads (3·3 % *v*. 1·9 %, *P* = 0·015).

**Table 2.** Median ferritin concentration and iron deficiency and iron deficiency anaemia prevalence among the Ethiopian population, 2015 (Median values)

Characteristics	*n*	Ferritin (µg l^−1^)		Iron deficiency (%)*	Iron deficiency anaemia (%)†
		Median	Q1, Q3		
Regions					
Addis Ababa	111	73·3	39·2, 115·2	4·6	2·6
Afar	160	53·9	32·4, 82·3	8·5	6·0
Amhara	343	71·4	49·5, 97·6	2·3	0·0
Benishangul-Gumuz	142	71·7	42·9, 102·8	3·5	2·5
Dire Dawa	111	47·7	25·5, 78·5	12·4	5·4
Gambela	115	43·1	22·9, 68·3	15·2	5·6
Harari	166	51·3	34·1, 75·6	4·0	2·0
Oromia	315	50·7	29·9, 77·2	7·6	3·7
SNNPR	208	55·4	31·0, 90·5	6·2	3·7
Somali	122	33·4	16·3, 59·8	23·2	14·6
Tigray	253	59·6	43·6, 89·9	4·3	1·2
National	2046	57·1	37·8, 89·0	5·8	2·6
Demographic group					
YC	338	37·5	18·6, 51·9	13·8	7·7
SAC	620	56·8	40·6, 78·7	3·3	1·0
Men	236	97·0	49·1, 143·8	10·6	2·3
WRA	852	66·1	43·6, 93·1	3·5	2·1
residence					
Urban	555	57·0	37·5, 90·5	3·6	2·1
Rural	1491	57·3	37·9, 88·6	6·2	2·8
Education status					
Household head is literate	1042	58·3	37·7, 93·4	5·7	1·9
Household head is illiterate	1004	56·7	38·3, 85·5	5·9	3·3

Q1, 25^th^ percentile; Q3, 75^th^ percentile; YC, young children; SAC, school-age children; WRA, women of reproductive age; ID, iron deficiency; IDA, iron deficiency anaemia.

Household heads attended elementary school or above were considered literate.

* ID defined as serum ferritin < 12 µg/l for young children and < 15 µg/l for school-age children, men and WRA.

† IDA defined as iron deficiency with anaemia.

### Intestinal parasitic infection

Intestinal helminthic parasites were detected in 38·3 % of the study groups. Highest infection rate was observed among SAC (49 %) followed by YC (47·2 %). Parasite infection was found in 39 % of men and 29·2 % of non-pregnant WRA. Cyst of *Giardia lamblia* (20·9 %), cyst of *Entamoeba histolytica* (18 %) and ova of *Ascaris lumbricoides* (5·1 %) were the predominant parasites identified.

### Association between serum micronutrients and Hb concentration

There was positive correlation between Hb and Ca (*r* = 0·24), Mg (*r* = 0·15), Zn (*r* = 0·27) and se (*r* = 0·15) concentrations (online Supplementary Table 3).

### Mediation effect of Zn on the association between se and Hb concentration

**Fig. 1.**
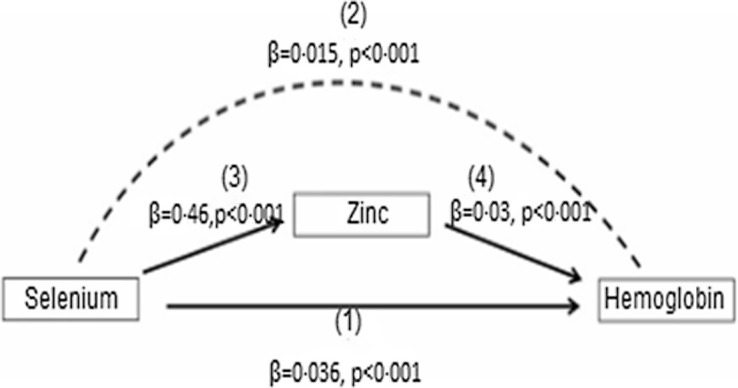
Mediation role of Zn on the effect of se on Hb concentrations among the Ethiopian population. se has both direct and indirect effect on Hb. The direct relation between se and Hb is shown by (1). The indirect effect of se on Hb, which is represented by the curved dash arrow (2) which is mediated by Zn with the effect of se on Zn (3) and the effect of Zn on Hb is indicated by the solid arrows (4). The mediation analysis was computed with the use of the Sobel–Goodman test.

In the present study, the Sobel–Goodman mediation test indicates that there was a significant partial mediation effect of Zn on the association between se and Hb. About 30 % of the effect of se on Hb was mediated by Zn (*P* < 0·001) (Fig. 1).

### Association between demographic characteristics and nutritional factors with Hb concentration

The multivariate analysis showed that age and serum ferritin concentration were significant predictors of Hb concentration in YC (Table 3), and a unit (year) increase in age is associated with a 0·19 (95 % CI 0·05, 0·33) g/dl increase in Hb. In addition, Hb concentration of YC increased by 0·01 (95 % CI 0·01, 0·02) g/dl with each increment of ferritin concentration. The multivariate regression shows that nutritional factors and non-nutritional factors predicted 6·1 and 3·3 % of the anaemia, respectively, among YC.

**Table 3.** Association between demographic factors, health and nutritional status with Hb concentration among Ethiopian young and school-age children, 2015 (95 % confidence intervals)

	Young children						School-age children					
Variable	Crude *β*	95 % CI	*P*	Adjusted *β*	95 % CI	*P*	Crude *β*	95 % CI	*P*	Adjusted *β*	95 % CI	*P*
Age, year	0·22	0·08, 0·35	0·001	0·19	0·04, 0·33	0·008	0·12	0·08, 0·15	< 0·001	0·08	0·05, 0·12	< 0·001
Sex												
Female	1						1					
Male	0·13	–0·12, 0·39	0·310	–		–	–0·04	–0·24, 0·17	0·732	–		–
Educational level												
Household head is illiterate	1						1					
Household head is literate	–0·01	–0·27, 0·25	0·932	–		–	0·25	0·05, 0·46	0·017	0·18	–0·02, 0·37	0·085
Residence												
Urban	1						1					
Rural	–0·03	–0·37, 0·31	0·861	–		–	–0·46	–0·71, −0·21	< 0·001	–0·22	–0·47, 0·03	0·090
Stool parasite												
No	1						1					
Yes	0·11	–0·14, 0·37	0·377	–		–	0·04	–0·16, 0·24	0·702			
CRP												
< 5 mg/l	1						1					
> 5 mg/l	–0·002	–0·03, 0·02	0·849	–		–	–0·05	–0·09, −0·003	0·038	–0·03	–0·07, 0·02	0·195
AGP												
< 1 g/l	1						1					
> 1 g/l	–0·23	–0·44, −0·02	0·034	–0·21	–0·43, 0·02	0·069	–0·17	–0·49, 0·14	0·284			
Diarrhoea												
No	1						1					
Yes	–0·32	–0·75, 0·11	0·146	–0·28	–0·69, 0·13	0·184	–0·49	–0·93, −0·04	0·034	–0·27	–0·69, 0·16	0·213
Coughing												
No	1						1					
Yes	–0·04	–0·39, 0·30	0·799	–		–	–0·35	–0·66, −0·04	0·028	–0·22	–0·52, 0·07	0·133
Fever												
No	1						1					
Yes	0·11	–0·26, 0·49	0·545	–		–	–		–	–		–
WAZ-score < –2	0·10	–0·01, 0·21	0·062	0·09	–0·02, 0·19	0·106						
WHZ-score < –2	–0·02	–0·12, 0·09	0·758	–		–	–		–	–		–
BAZ-score < –2	–		–	–		–	0·04	–0·04, 0·13	0·309	–		–
Serum ferritin	0·01	0·01, 0·02	< 0·001	0·01	0·01, 0·02	< 0·001	0·01	0·02, 0·01	< 0·001	0·01	0·001, 0·01	0·002
Serum Ca	0·10	–0·07, 0·29	0·264	–		–	0·15	0·00, 0·30	0·051	0·06	–0·11 023	0·470
Serum Mg	0·35	–0·29, 0·99	0·283	–		–	0·34	–0·20, 0·88	0·219	–		–
Serum se	0·01	–0·02, 0·05	0·443	–		–	0·02	–0·01, 0·04	0·152	0·03	0·00, 0·05	0·050
Serum Zn	0·01	–0·01, 0·02	0·465	–		–	0·03	0·02, 0·04	< 0·001	0·02	0·01, 0·05	< 0·001
Serum Cu	–0·001	–0·01, 0·01	0·965	–		–	–0·01	–0·01, −0·001	0·022	–0·004	–0·01, 0·001	0·162
Serum Mo	–0·31	–1·77, 1·15	0·675	–		–	–0·77	–1·81, 0·27	0·148	–0·65	–1·73, 0·42	0·234
Serum Co	–0·45	–5·50, 4·54	0·852	–		–	–6·75	–10·58, −2·93	0·001	–3·23	–7·07, 0·60	0·099

WAZ, weight-for-age z-score; WHZ, weight-for-height z-score; BAZ, BMI-for-age z-score; CRP, C-reactive protein; AGP, *α*-1 acid glycoprotein.

The results were generated based on bivariate and multivariate regression statistical tests. Household heads attending elementary school or above were considered literate or illiterate otherwise.

Using multilevel logistic regression analysis, the main predictors of anaemia in YC were age, being underweight and low serum ferritin (Table 4). YC who were underweight were three times (adjusted OR: 2·85; 95 % CI 1·37, 5·91) more likely to be anaemic than normal-weight children. Also, YC with low serum ferritin were ten times more likely (adjusted OR: 10·32; 95 % CI 5·04, 21·11) to be anaemic compared with YC with normal serum ferritin concentration.

**Table 4.** Association between nutrition and health status with anaemia incidence among young (6–59 months) and school-age children (6–14 years) in Ethiopia, 2015 (Odds ratios; 95 % confidence intervals)

Variable	Young children			School-age children		
	Adjusted OR	95 % CI	*P*	Adjusted OR	95 % CI	*P*
Age, year	0·61	0·44, 0·83	0·002	0·86	0·79, 0·92	< 0·001
WAZ-score < –2	2·85	1·37, 5·91	0·005	–		–
Low serum ferritin (adjusted)	10·32	5·04, 21·11	< 0·001	2·81	1·32, 5·99	0·007
AGP, > 1g/l	1·06	0·55, 2·32	0·863	–		–
Diarrhoea	0·90	0·30, 2·71	0·854			
Household head is illiterate	–		–	0·64	0·42, 0·98	0·042
Residence, rural	–		–	1·09	0·62, 1·91	0·759
Coughing, yes	–		–	1·23	0·69, 2·19	0·472
CRP, > 5 mg/l	–		–	1·82	0·79, 4·18	0·155
Low serum Co	–		–	0·44	0·19, 0·97	0·042
Low serum se	–		–	1·43	0·93, 2·19	0·099
Low serum Cu	–		–	1·21	0·53, 2·74	0·652
Low serum Zn	–		–	1·78	0·85, 3·69	0·122

WAZ, weight-for-age z-score; CRP, C-reactive protein; AGP, *α*-1 acid glycoprotein.

The results were generated based on logistic regression statistical analysis.

Table 3 shows results of the bivariate and multivariate regression analyses for SAC. The main predictors of Hb in SAC were age and ferritin, se and Zn concentration in the serum. The regression analysis also shows that nutritional factors and non-nutritional factors predicted 7·9 and 7·3 % of the anaemia incidence, respectively, among SAC.

The main predictors of anaemia in SAC were age, household head illiteracy, low serum ferritin concentration and high serum Co concentration (Table 4). A one-year increase in the age of SAC was associated with a 14 % (adjusted OR: 0·86; 95 % CI 0·79, 0·92) reduction in anaemia occurrence. SAC in households having literate household heads had 36 % (adjusted OR: 0·64; 95 % CI 0·42, 0·98) lower odds of being anaemic in comparison with those SAC in households whose heads were illiterate. Similarly, SAC with low serum ferritin concentration were 2·8 times (adjusted OR; 2·81; 95 % CI 1·32, 5·99) more likely to be anaemic than those with normal ferritin concentration. Furthermore, SAC who had low serum Co concentration were 56 % (adjusted OR: 0·44; 95 % CI 0·19, 0·97) less likely to be anaemic than their normal counterparts.

Results of multivariate linear regression analysis (Table 5) show that Hb concentration had a significant positive association with serum Zn and household head literacy but was negatively associated with serum Cu concentration, AGP concentration and presence of diarrhoea. In addition, household head illiteracy, presence of diarrhoea, low serum Zn and Cu concentrations were significant predictors of Hb among men. Among mineral micronutrients, only low serum Cu was a predictor of anaemia in men (Table 6).

**Table 5.** Association between social, health and nutritional characteristics with Hb concentration among Ethiopian men and non-pregnant women of reproductive age, 2015 (95 % confidence intervals)

Variable	Men						Non-pregnant women of reproductive age					
	Crude *β*	95 % CI	*P*	Adjusted *β*	95 % CI	*P*	Crude *β*	95 % CI	*P*	Adjusted *β*	95 % CI	*P*
Age, year	0·001	–0·01, 0·01	0·936	–		–	–0·01	–0·02, −0·003	0·008	–0·01	–0·02, 0·004	0·177
Educational level												
Household head is illiterate	1						1					
Household head is literate	0·59	0·16, 1·01	0·007	0·54	0·12, 0·96	0·013	0·42	0·26, 0·59	< 0·001	0·28	0·05, 0·51	0·019
Residence												
Urban	1						1					
Rural	–0·70	–1·11, −0·28	0·001	–0·33	–0·77, 0·127	0·158	–0·27	–0·45, −0·09	0·003	–0·01	–0·25, 0·23	0·948
Stool parasite												
No	1						1					
Yes	0·07	–0·31, 0·46	0·705	–		–	0·004	–0·19, 0·19	0·970	–		–
CRP												
< 5 mg/l	1						1					
> 5 mg/l	–0·02	–0·05, 0·01	0·243	–		–	0·001	–0·02, 0·02	0·964	–		–
AGP												
< 1 g/l	1						1					
> 1 g/l	–0·70	–1·30, −0·11	0·020	–0·75	–1·38, −0·11	0·021	–0·14	–0·43, 0·16	0·360	–		–
Diarrhoea												
No	1						1					
Yes	–0·19	–1·09, 0·71	0·673	–		–	–0·13	–0·50, 0·25	0·512	–		–
Coughing												
No	1						1					
Yes	–0·44	–0·97, 0·10	0·112	–0·31	–0·82, 0·21	0·248	0·004	–0·27, 0·28	0·974	–		–
Fever												
No	1						1					
Yes	0·05	–0·53, 0·63	0·862				–0·19	–0·45, 0·07	0·156	–0·06	–0·35, 0·22	0·653
BMI												
Normal	1						1					
Wasting	–0·48	–0·87, −0·09	0·017	–0·45	–0·84, −0·062	0·023	–0·11	–0·33, 0·11	0·320	–0·03	–0·27, 0·21	0·817
Over weight	0·49	–0·24, 1·24	0·187	0·25	–0·49, 0·99	0·512	0·23	–0·03, 0·49	0·083	0·15	–0·18, 0·49	0·369
Serum ferritin	0·001	–0·004, 0·002	0·557	–		–	0·01	0·003, 0·01	< 0·001	0·005	0·002, 0·007	< 0·001
Serum Ca	0·28	–0·07, 0·62	0·114	0·05	–0·29, 0·39	0·755	0·09	–0·04, 0·24	0·177	–0·01	–0·20, 0·19	0·948
Serum Mg	0·16	–1·07, 1·38	0·800	–		–	0·39	–0·08, 0·86	0·106	0·04	–0·56, 0·65	0·759
Serum se	0·05	0·01, 0·09	0·030	0·01	–0·04, 0·06	0·699	0·02	–0·002, 0·04	0·074	–0·003	–0·03, 0·02	0·979
Serum Zn	0·02	0·01, 0·04	0·009	0·02	0·001, 0·04	0·040	0·02	0·01, 0·03	< 0·001	0·02	0·01, 0·03	0·002
Serum Cu	–0·01	–0·02, 0·001	0·084	–0·01	–0·02, −0·001	0·026	–0·001	–0·005, 0·003	0·725	–		–
Serum Mo	0·44	–1·71, 2·59	0·687	–		–	–0·42	–1·44, 0·59	0·417	–		–
Serum Co	–4·71	–11·27, 1·85	0·159	–0·29	–6·29, 7·03	0·914	–3·53	–6·58, −0·48	0·023	0·67	–3·076, 4·40	0·727
Serum folate	–		–	–		–	0·02	0·01, 0·03	< 0·001	0·02	0·01, 0·02	< 0·001

CRP, C-reactive protein; AGP, *α*1-acid glycoprotein.

Household heads attending elementary school or above were considered literate or illiterate otherwise. The results were generated based on bivariate and multivariate regression statistical tests.

**Table 6.** Association between social and nutritional characteristics and anaemia incidence among Ethiopian men (15–54 years) and non-pregnant women of reproductive age, 2015 (Odds ratios and 95 % confidence intervals)

Variable	Men			Non-pregnant women of reproductive age		
	Adjusted OR	95 % CI	*P*	Adjusted OR	95 % CI	*P*
Household head is literate	1·01	0·46, 2·16	0·988	0·54	0·31, 0·95	0·032
Residence, rural	1·43	0·58, 3·52	0·434	–		–
AGP, > 1 g/l	1·63	0·76, 3·51	0·321	–		–
Wasting	1·61	0·79, 3·25	0·181	–		–
Over weight	1·24	0·72, 2·15	0·427	–		–
Low serum Cu	0·12	0·02, 0·90	0·040	–		–
Low serum Zn	0·88	0·36, 2·14	0·781	1·33	0·64, 2·78	0·444
Age, year	–		–	1·00	0·97, 1·04	0·745
Low ferritin	–		–	8·51	3·96, 18·3	< 0·001
Low serum folate	–		–	2·74	1·49, 5·03	0·001

AGP, *α*-1 Acid glycoprotein.

Household heads attending elementary school or above were considered literate or illiterate otherwise. The results were generated based on bivariate and multivariate regression statistical tests.

Table 5 shows the association between social, health and nutritional characteristics with Hb concentration in WRA. The result shows that household head literacy, serum ferritin, serum Zn and serum folate concentrations were significant predictors for Hb concentration. A positive association between serum Zn (*β* = 0·02; 95 % CI 0·01, 0·03), ferritin (*β* = 0·004; 95 % CI 0·002, 0·007) and folate (*β* = 0·02; 95 % CI 0·01, 0·02) with Hb was observed. Average Hb concentration of WRA of households headed by a literate person was 0·28 g/dl (95 % CI 0·04, 0·51) greater compared with women from households headed by an illiterate person.

Table 6 shows results of the multilevel logistic regression analysis of the association between nutritional and non-nutritional factors with anaemia incidence among WRA. Accordingly, the main risk factors were household head illiteracy, low serum ferritin and folate concentration. Women from households with literate household heads were 46 % (adjusted OR: 0·54; 95 % CI 0·31, 0·95) less likely to be anaemic than their counterparts. Also, women with low serum ferritin concentration were 8-fold (adjusted OR: 8·51; 95 % CI 3·96, 18·3) more likely to be anaemic than those with sufficient serum ferritin concentration. In addition, women with low serum folate were 2·7-fold (adjusted OR: 2·74; 95 % CI 1·49, 5·03) more likely to be anaemic compared with women with serum folate concentration in the normal range.

## Discussion

Few studies have examined the association between multiple micronutrient status and socio-demographic factors, and anaemia in developing^(7,15,23,59)^ and developed country settings^(8,60)^, partly because few studies measure Hb along with such a wide range of factors. In this population-based cross-sectional study, nutritional and non-nutritional factors were main predictors of anaemia incidence among different demographic groups.

Serum ferritin is a main predictor of anaemia in all demographic groups except men. It is a good biomarker to assess Fe status in the population, because it reflects the concentration of stored Fe in the liver^(61)^, but it is affected by infection, so ferritin concentration should be corrected for inflammation when estimating the prevalence of ID^(44)^. In this study, low ferritin was a predictor of anaemia in YC, SAC and WRA, indicating that low serum ferritin increased the odds of developing anaemia which is consistent with the fact that ID can cause anaemia^(4)^.

In the present study, Zn was a main predictor of Hb in all demographic groups except YC, but not a determining factor of anaemia, consistent with findings in Vietnam among SAC and adolescent girls^(15,16)^. Other studies among pregnant women in Ethiopia^(62)^, YC in USA^(60)^, women in Malawi^(63)^ and SAC in New Zealand^(8)^ have reported that Zn is a predictor of anaemia. Zn may affect Hb via several Zn-dependent enzyme systems involved in Hb synthesis and stabilising red cell membranes^(10,11,64,65)^. Under Zn deficiency, the lifespan of erythrocytes is shortened because of the reduced activity of erythrocyte Cu-Zn superoxide dismutase, which is a Zn-dependent enzyme that protects against oxidative stress and contributes to cell integrity^(66)^.

Co is involved in haematopoiesis and vitamin B_12_ synthesis^(67)^. In this present study, Co is inversely associated with Hb in SAC consistent with findings among Swedish adolescent girls^(68)^, Norwegian boys^(69)^, pregnant women in Spain^(34)^ and Japanese children^(7)^. This may be because Co absorption is mediated by the divalent metal transporter 1, which is up-regulated by Fe status^(33)^. Similar to other divalent metals, a decrease in Fe status may trigger divalent metal transporter 1 expression, resulting in an increase in Co absorption.

A weak negative correlation was found between Hb–Co, Hb–Cu and Hb–Mo, while a positive correlation was found between Hb–Ca, Hb–Mg, Hb–Zn and Hb–se pairs (online Supplementary Table 3). However, these correlations can only be exploratory and should be interpreted with caution. The bivariate and multivariate regression analysis shows that se is a predictor of Hb in SAC as reported by other studies^(13–17,70)^. The Hb–se association is supported by our mechanistic understanding that se has a role in the regulation of hepatic heme oxygenase-1 activity. A low concentration of se can cause the up-regulation of the heme oxygenase-1 enzyme, which facilitates the heme catabolism, leading to a depletion of heme^(71)^.

The present study data show that the association of se with Hb was partially mediated through serum Zn, as shown by significant direct association se–Hb, serum se–Zn and serum Zn–Hb path coefficients. In a mediation model, the effect of an independent variable (se) on a dependent variable (Hb) is transmitted through Zn as depicted in Fig. 1. This finding may be due to low se status affecting glutathione peroxidase activity, a selenoprotein that regulates the release and transfer of Zn from metallothionein to Cu–Zn superoxide dismutase^(20)^. This finding indicates the direct effect of se deficiency on Hb concentration and indirectly compromises human Zn status suggesting the importance of addressing se deficiency for normal Zn metabolism and Hb synthesis.

Serum Cu was negatively associated with Hb and was a predictor of anaemia among the present study men. In Japanese children, no association between Cu and anaemia was observed^(7)^, but a low level of Cu was observed in the blood of anaemic YC and SAC in Pakistan^(72)^. Furthermore, a study in US adults revealed that serum Cu levels outside the normal range (both low and high) were positively associated with anaemia^(31)^. This is because Cu is essential for the functioning of many Cu-dependent enzymes^(73)^, such as ceruloplasmin which is responsible for antioxidant protection, Fe metabolism and Cu transportation. Anaemia appears due to the defects in Fe mobilisation as a result of combined defect of ceruloplasmin ferroxidase activity and intracellular utilisation^(74–76)^.

Folate is a water-soluble vitamin which acts as a co-enzyme in various organic reactions^(77)^. Studies have found that low level of folate in WRA can be a risk factor for anaemia^(78,79)^. This is because folate deficiency has many consequences, such as megaloblastic anaemia, resulting from ineffective erythropoiesis which leads to decreased erythrocyte production and subsequently to decreased numbers of circulating erythrocytes^(80)^.

Other predictors of anaemia include underweight, household head illiteracy and age. For example, 38·4 % of underweight children were anaemic. The studies in Indian^(81)^ and Iranian^(82)^ adolescent girls show that anaemia is not significantly associated with BMI status. Conversely, other studies have shown high prevalence of anaemia in undernourished children and adults of both sexes with low WAZ-score and BMI (< 18·5 kg/m^2^), respectively^(17,83–88)^. In Lao People’s Democratic Republic, national representative samples showed that underweight children (WAZ score < –2 sd) were more likely to be anaemic than non-underweight children^(84)^. This may be because inadequate dietary diversity or energy consumption may affect the nutritional status of children and Hb synthesis^(89,90)^.

Our study found that SAC and non-pregnant WRA participants in households where the head household member (half of the household heads in the present study were females) had completed primary and higher education levels were less likely to be anaemic in comparison with the households where the head is illiterate. In Korean children, a study shows that maternal education independently affected the likelihood of children developing anaemia and/or ID^(91)^. A study in Ethiopian children also reported that one of the risks of anaemia is maternal illiteracy^(92)^. Maternal education affects children’s diets and has a direct influences on children’s health^(93–95)^. This may be because low level of education can lead to less access to diverse diets^(94)^.

Age was a predictor of anaemia in YC and SAC in Ethiopia. A previous study in Ethiopia found that age was not a determinate factor for anaemia in SAC^(96)^ nor among children aged under five in Tanzania^(97)^. However, other studies have reported that anaemia decreased with increasing age among children aged under five in Bangladesh^(98)^ and among Ethiopian children aged 6–23 months^(99)^. Anaemia might be associated with poor infant and young child feeding practices whereby complementary foods lack adequate Fe and other micronutrients increasing risk of anaemia^(100)^.

In the present study, nutritional factors (minerals and vitamins) associated with anaemia are minimal. In addition, IDA was low and inflammation was not associated with anaemia indicating the presence of other factors not captured in this study. Thus, further studies investigating other risk factors such as haemoglobinopathies, water quality and sanitation facilities are warranted.

A strength of our study is a large sample size, the inclusion of different demographic groups, the simultaneous analysis of serum micronutrients conducted with highly sensitive instruments and the application of multivariate regression models to elucidate the contribution of multiple factors to anaemia risks, as well as the mediation analysis of serum Zn on the association of serum se and Hb. A weakness of the study is the lack of data on detailed dietary intakes of the participants and other measures of socio-economic status. Furthermore, the nature of the study design, that is, cross-sectional, limits us to observing associations and does not allow us to determine the causality of factors that affect the Hb status of the study groups. The present study is limited to detecting the presence and identification of intestinal parasites in stool samples. However, parasitic load, an important factor of anaemia, was not quantified.

### Conclusion

The present study determined the magnitude of anaemia among different demographic groups of the Ethiopian population. It also tested the association between nutritional, demographics, health condition and household characteristics with Hb concentration and identified the potential factors for the prevalence of anaemia. In general, about 19 % of the population in Ethiopia were anaemic, 6 % had ID and 3 % had IDA. In addition, 0·6 % had tissue ID. In the present study subjects, low serum ferritin, folate, se Zn and Cu, older age, household head illiteracy and low BMI were the significant determinants of anaemia. There was also a significant mediation effect of Zn on the positive association between se and Hb.

This is a unique and large national scale study reporting anaemia incidence among different demographic groups in the country. Because anaemia is multifactorial and the present study identified the most important factors influencing Hb concentration, the result will be important to design a multi-sectorial approach (adult literacy/nutrition education, maternal folate supplementation programmes and micronutrient deficiency alleviation programmes), prioritising nutrition and health programmes to address risks of anaemia.

## Supplementary material

For supplementary material accompanying this paper visit https://doi.org/10.1017/S0007114523001472. click here to view supplementary material
