# Supplementary material for: Micronutrients and socio-demographic factors were major predictors of anaemia among the Ethiopian population
Source: Br J Nutr. 2023 Jul 10;130(12):2123–35. doi: 10.1017/S0007114523001472 (PMC10657751; doi:10.1017/S0007114523001472)
Supplement: Supplementary file 1 [file S0007114523001472sup.zip › S0007114523001472sup002.docx]

**Supplementary Table 1.** Hemoglobin concentrations and prevalence of Anemia by different variable

| **Variable** | **n** | **Hb (g dL^-1^), Median [Q1, Q3]** | **Prevalence of anemia ¶** |
| --- | --- | --- | --- |
| **Gender** |  |  |  |
| Female | 1348 | 13.1 [12.2,13.9] | 16.7 |
| Male | 698 | 13.0 [12.0,14.3] | 22.2 |
| **Stool parasite** |  |  |  |
| No | 1266 | 13.1 [12.1,14.2] | 19.8 |
| Yes | 780 | 13.0 [12.2,13.9] | 16.9 |
| **CRP** |  |  |  |
| <5 mg L^-1^ | 1846 | 13.1 [12.1,14.1] | 18.5 |
| >5 mg L^-1^ | 198 | 12.7 [12.1,13.8] | 19.6 |
| **AGP** |  |  |  |
| <1 g L^-1^ | 1502 | 13.1 [14.2,15.0] | 17.5 |
| >1 g L^-1^ | 544 | 12.8 [11.9,13.7] | 21.5 |
| **Diarrhoea** |  |  |  |
| No | 1922 | 13.1 [12.1,14.1] | 18.4 |
| Yes | 124 | 12.5 [11.7,14.3] | 21.1 |
| **Coughing** |  |  |  |
| No | 1788 | 13.1 [12.1,14.1] | 18.4 |
| Yes | 258 | 13.0 [12.0,14.1] | 20.3 |
| **Fever** |  |  |  |
| No | 1817 | 13.0 [12.1,14.1] | 19.2 |
| Yes | 229 | 13.3 [12.3,14.4] | 12.5 |
| **Nutritional status** |  |  |  |
| Normal | 748 | 13.6 [12.7,14.5] | 10.9 |
| Wasting | 1168 | 12.7 [11.8,13.6] | 23.8 |
| Over weight | 130 | 13.5 [12.6,14.4] | 12.5 |
| Normal serum Ferritin (above Cut-off) | 1889 | 13.1 [12.2,14.1] | 16.8 |
| Low serum Ferritin (below cut-off) | 157 | 11.9 [10.8,13.2] | 48.0 |
| Normal serum calcium (above Cut-off) | 1191 | 13.3 [12.4,14.3] | 17.1 |
| Low serum calcium (below cut-off) | 855 | 12.7 [11.8,13.6] | 20.7 |
| Normal serum magnesium (above Cut-off) | 1521 | 13.3 [12.3,14.2] | 17.5 |
| Low serum magnesium(below cut-off) | 525 | 12.7 [11.7,13.7] | 21.3 |
| Normal serum selenium (above Cut-off) | 1457 | 13.3 [12.3,14.3] | 16.5 |
| Low serum selenium(below cut-off) | 589 | 12.8 [11.8,137] | 21.8 |
| Normal serum zinc (above Cut-off) | 291 | 13.5 [12.6,14.6] | 15.2 |
| Low serum zinc(below cut-off) | 1755 | 13.0 [12.0,14.0] | 19.1 |
| Normal serum copper (above Cut-off) | 1919 | 13.1 [12.1,14.1] | 18.4 |
| Low serum copper (below cut-off) | 127 | 12.7 [11.9,13.8] | 21.4 |
| Normal serum molybdenum (above Cut-off) | 2036 | 13.0 [12.1,14.1] | 18.5 |
| Low serum molybdenum(below cut-off) | 10 | 12.3 [11.7,12.8] | 27.6 |
| Normal serum cobalt (above Cut-off) | 1705 | 13.0 [12.1,14.0] | 19.5 |
| Low serum cobalt(below cut-off) | 341 | 13.3 [12.4,14.4] | 13.3 |

**¶**Anemia cut-off for young children < 11.0 g g dL^-1^, <11.5 g dL^-1^for children 5-11 years, <12.0 g dL^-1^for children 12-14 years, <12.0 g dL^-1^ for WRA and <13.0 g dL^-1^ for men.

**Supplementary Table 2.** sTfR concentration and prevalence of iron and folate deficiency by study characteristics

| Characteristics | n | sTfR (mg L^-1^), Median [Q1,Q3] | Prevalence of Iron deficiency  (%)* |
| --- | --- | --- | --- |
| **Regions** |  |  |  |
| Addis Ababa | 111 | 3.4 [2.9,3.8] | 0.0 |
| Afar | 160 | 2.8 [1.7,3.8] | 0.5 |
| Amhara | 343 | 3.0 [2.5,3.4] | 0.6 |
| Benishagul-Gumuz | 142 | 3.3 [2.8, 3.8] | 1.7 |
| Dire Dawa | 111 | 3.5 [2.8,4.2] | 1.5 |
| Gambela | 115 | 3.6 [3.0,4.2] | 1.2 |
| Harari | 166 | 2.7 [2.3,3.0] | 0.0 |
| Oromia | 315 | 3.2 [2.4,3.8] | 0.3 |
| SNNPR | 208 | 2.6 [1.9,3.4] | 0.9 |
| Somali | 122 | 3.0 [2.4,3.7] | 3.5 |
| Tigray | 253 | 2.5 [2.3,2.8] | 0.9 |
| **National** | 2,046 | 2.9 [2.4,3.5] | 0.6 |
| **Demographic group** |  |  |  |
| YC | 338 | 3.1 [2.5,3.8] | 0.1 |
| SAC | 620 | 3.1 [2.6,3.6] | 0.4 |
| Men | 236 | 2.7 [2.1,3.5] | 2.3 |
| WRA | 852 | 2.7 [2.1,3.3] | 0.6 |
| **Residence** |  |  |  |
| Urban | 555 | 3.0 [2.4,3.5] | 0.4 |
| Rural | 1,491 | 2.9 [2.4,3.6] | 0.7 |
| **Education status** |  |  |  |
| Household head is Educated | 1,042 | 2.9 [2.3,3.6] | 0.4 |
| Household head is illiterate | 1,004 | 2.9 [2.4,3.5] | 0.9 |

*Iron deficiency defined as serum sTfR> 4.4 mg L^-1^ for young children, school age children and WRA, and > 5 mg L^-1^ for men

**Supplementary Table 3** Pearson’s rank correlations between Hb and minerals of all demographic groups

| **Indicators** | **Hb** | **Ca** | **Mg** | **Co** | **Cu** | **Zn** | **Se** | **Mo** |
| --- | --- | --- | --- | --- | --- | --- | --- | --- |
| Hb | 1.00 |  |  |  |  |  |  |  |
| Ca | 0.24 | 1.00 |  |  |  |  |  |  |
| Mg | 0.15 | 0.65 | 1.00 |  |  |  |  |  |
| Co | -0.10 | 0.04 | 0.03 | 1.00 |  |  |  |  |
| Cu | -0.11 | 0.15 | 0.21 | -0.04 | 1.00 |  |  |  |
| Zn | 0.27 | 0.47 | 0.34 | -0.05 | 0.11 | 1.00 |  |  |
| Se | 0.15 | 0.18 | 0.16 | -0.05 | 0.08 | 0.18 | 1.00 |  |
| Mo | -0.06 | -0.08 | 0.00 | -0.02 | 0.01 | -0.09 | 0.33 | 1.00 |
