# Supplementary material for: Micronutrients and socio-demographic factors were major predictors of anaemia among the Ethiopian population
Source: Br J Nutr. 2023 Jul 10;130(12):2123–35. doi: 10.1017/S0007114523001472 (PMC10657751; doi:10.1017/S0007114523001472)
Supplement: Supplementary file 1 [file S0007114523001472sup.zip › S0007114523001472sup001.docx]

**Supplementary Figure 1.** Mediating role of zinc within the effect of selenium on hemoglobin concentrations in Afar, Oromia, Amhara, Somali, SNNP and Tigray Regions. The entire effect of selenium on hemoglobin comprised direct and indirect effects. The direct relation between selenium and hemoglobin is shown by (1). The indirect effect of selenium on hemoglobin, which is represented by the curved dashed arrow (2) which is mediated by zinc with the effect of selenium on zinc (3) and the effect of zinc on hemoglobin shown by the solid arrows (4). The mediation analysis was performed with the use of the Sobel-Goodman test.

**
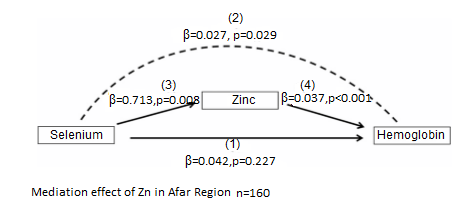
**

**
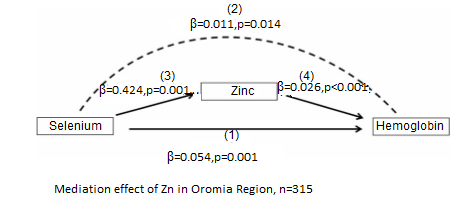
**

**
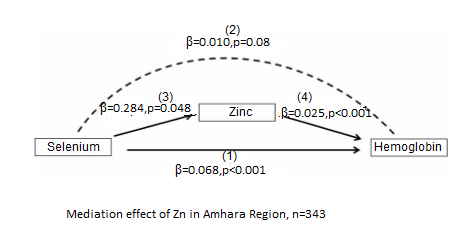
**

**
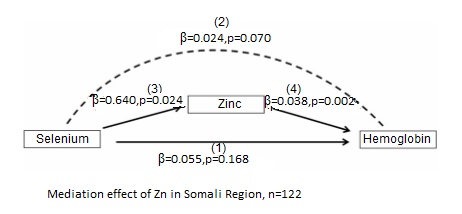
**

**
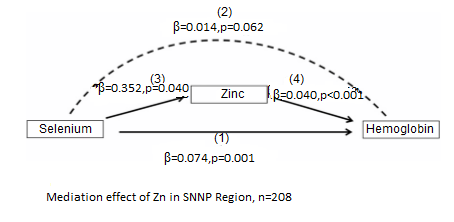
**

**
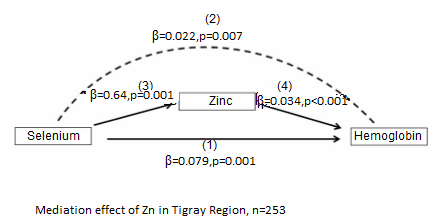
**
